# Evaluation of Enzyme Agarose Gels for Cleaning Complex Substrates in Cultural Heritage

**DOI:** 10.3390/gels10010014

**Published:** 2023-12-22

**Authors:** Mattia Morlotti, Fabio Forlani, Ilaria Saccani, Antonio Sansonetti

**Affiliations:** 1 Brera Academy of Fine Arts/Freelance Conservator of Cultural Heritage, 20100 Milan, Italy; mattiamorlotti@fadbrera.edu.it; 2 Department of Food Environmental and Nutritional Science (DeFENS), University of Milan, 20133 Milan, Italy; fabio.forlani@unimi.it; 3 CESMAR7—Centro per lo Studio dei Materiali per il Restauro, 42121 Reggio Emilia, Italy; saccani.ila@gmail.com; 4 Institute for Heritage Science, National Research Council, ISPC—CNR Milan Unit, 20154 Milan, Italy

**Keywords:** enzyme gels, enzymatic cleaning, cleaning evaluation, trypsin, lipase, agarose gels, DCA-TEA

## Abstract

This study starts from the need to remove a mix of proteins, oils and natural resin, called *beverone* in the Italian literature, from the back of canvas paintings. The aim of this study is to develop and evaluate the effectiveness of two different agarose/enzyme gels containing, respectively, a trypsin derived from porcine pancreas and a lipase from *Candida rugosa*, both in an aqueous solution of deoxycholic acid-triethanolamine soap. Enzymes were selected because of their action on peptide and ester bonds, effectiveness at maintaining a weak alkaline pH and low cost. Several series of model samples, resulting from a combination of rabbit skin glue, linseed oil and colophony, were prepared to test the enzyme gels with two different values for each of the following variables: agarose concentration, application modes and time of application. Measurements of weight loss after the gel application and Fourier transform infrared analysis were conducted to underline the hydrolysis occurring due to the enzyme gels and their effectiveness. Results confirmed what has been found in the literature and improved our knowledge about the action of agarose enzyme gels on complex substrates (hydrophilic/hydrophobic). The gels applied fluidly, with a longer contact time and a lower agarose concentration, are more effective. Furthermore, trypsin gels provided better results on substrates with oil and glue, while lipase gels turned out to be more effective on substrates made of a mix of oil, glue and colophony.

## 1. Introduction

Complex mixtures of natural organic substances have often been used by conservators on the back of canvas paintings in order to fix detachments and to revive the colors. The practice was in use particularly between the XVIII and XIX centuries. In Italy, the term *beverone* was used to indicate a general mix and each conservator in the past used their own personal recipe, normally a mix of animal glue, oils and/or natural resins. After several decades of decay, very often the presence of this application, which could be quite thick, caused a severe stiffening, a diffused darkening and even strong depolymerization of the canvas [1,2,3,4,5,6,7]. The resulting conservation problem requires safe and effective removal methods.

In this paper, the main results of a research project are presented, starting from a case study in which a painting on canvas presents a thick and stiff layer on the backside, possibly associated with the use of a *beverone* (Giuseppe Vermiglio, Head of St. Paul—first half of the XVII century) [8]. An analytical protocol was carried out to characterize this specific layer: FTIR analysis highlighted the presence of proteinaceous and terpene substances; GC-MS analysis confirmed the co-presence of colophony and linseed oil; meanwhile, an enzyme immuno-assay was able to distinguish the origin of the protein, indicating the presence of rabbit skin glue. The co-presence of hydrophilic (rabbit skin glue) and lipophilic fractions (linseed oil and colophony) suggested the use of an emulsion as a cleaning tool. It was decided to remove the *beverone* using an agar-agar gel and a weak alkaline solution of a deoxycholic acid–triethanolamine affinity soap (DCA-TEA) (Deoxycholic acid, DCA buffered with triethanolamine, TEA (pH 8.5), to form an affinity soap, specific for terpenic varnish layer removal) [9,10,11] loaded with a low percentage of benzyl alcohol. The mobility of the chemicals used is minimized by being entrapped in the gel network, allowing the end user to save solvents and other substances that are potentially hazardous to the environment. The application of a “green chemistry” approach can offer a valid alternative to traditional solvents and mixtures used for cleaning. For these reasons, the cleaning tests presented in this paper were performed using a gel formulation based on agarose and enzymes to obtain a “greener” solution. Agar-agar extracted from red algae is a natural mix of agarose and agaropectin and it may show different performances according to its provenance and manufacturing [12,13,14]. Agarose is purer and more homogeneous; for this reason, it is supposed to show more reproducible results [15]. Agarose is a natural polymer consisting of D-galactose and 3,6-anhydro-L-galactose repeating units. It is soluble in hot water, forming a gel after cooling below 45 °C because of extensive hydrogen bonding among the agarose chains [16]. The Ewaglos project [17] defined bio-cleaning as a tool using living organisms or their enzymes as cleaning agents, performing their natural metabolic processes in controlled conditions. Enzyme cleaning is a topic of growing interest and is based on biological reactions occurring in natural habitats, which are optimized in artificial conditions with the aim of cultural heritage conservation [18]. In the recent literature, several case studies of cultural heritage biotechnologies presented the removal of inorganic salts from stone substrate, as well as the removal of more complex inorganic matrixes such as black crusts [19,20], in addition to altered organic substances from frescoes [21]; on the other hand, enzyme cleaning used with the aim of removing such a complex organic matrix as beverone is quite unusual, especially when considering that enzymes can be put into contact with the soiled surface through the aid of a gelling system.

### Research Aims

“When new cleaning solutions are proposed, the conservation scientists are called to test them and assess their effectiveness on mock up samples, prior their application directly on real works of art” [22].

This paper deals with two main issues:

1. Tailoring of effective green gels, user-friendly and low cost, avoiding toxic organic solvents.
2. Set up of a testing protocol based on an analytical approach to evaluate and compare the cleaning effectiveness of different enzymatic gels.

In order to obtain these results, agarose gels loaded with two different enzymes were used on different substrates, modifying the following for each gel: (a) agarose concentration, (b) application method and (c) timing (Table 1). The term substrate is used in this paper to indicate a mockup prepared “ad hoc” to mimic the real painting with all the issues useful to test the cleaning method adopted here (see Section 2.2). For each cleaning parameter, only two values have been chosen, limiting the number of possible combinations. The pH value and temperature (8.5 and 37 °C) have not been considered as variables in this research and were kept constant during the gel application. DCA-TEA solution and a 37 °C temperature are commonly used in the field of conservation and no references in the literature provide a warning about interaction with the gel stability. Both the scientific literature [23,24,25,26,27,28,29] and the needs of conservation practice were considered for the testing set up.

## 2. Materials and Methods

### 2.1. Gel Materials

The gel polymer was a molecular biology-grade agarose (10766834, Fisher Scientific Italia, Segrate, MI, Italy). Enzymes selected were *C. rugosa* lipase (≥700 unit/mg solid; L1754, Merck KGaA, Darmstadt, Germany) and porcine pancreas trypsin (1000–2000 units/mg solid; T4799, Merck KGaA, Darmstadt, Germany). According to product specifications, one enzyme unit of lipase hydrolyzes 1 is the microequivalent of a fatty acid from a triglyceride in 1 h at a pH of 7.2 at 37 °C, and one enzyme unit of trypsin produces a 0.001 absorbance variation at 253 nm every min at a pH of 7.6 at 25 °C using Na-benzoyl-L-arginine ethyl ester (BAEE) as the enzyme substrate. The affinity soap solution (DCA-TEA) was composed of 4.7 g/L deoxycholic acid (Merck KGaA, Darmstadt, Germany), 1 mL/L triethanolamine (TEA; C.T.S. S.r.l., Altavilla Vicentina, VI, Italy) and adjusted to a pH of 8.5 (HCl). In the prepared DCA-TEA solution, the final concentrations were ~12 mM DCA and ~75 mM TEA.

A trypsin and a lipase were chosen to match the “green gel” requirements as defined in the previous paragraph; they are supposed to be active in particular on rabbit skin glue and linseed oil. For the specific substrates with linseed oil, Lipase type VII from *C. rugosa* was chosen. It has already been used several times in the field of conservation [30,31,32], also in combination with affinity soaps [33,34,35]. The choice of the enzyme to be used on the rabbit skin glue was more complicated because of the variety of several different enzymes suggested by the literature [36,37,38]. Considering the request for low cost and effectiveness, bacterial, fungal proteases and collagenase (Protease from *Aspergillus sojae* Type XIX, from *Aspergillus oryzae*, from *Aspergillus melleus* Type XXIII, Bacterial Type XXIV, from *Streptomyces griseus* Type XIV, Collagenase from Clostridium Histolyticum Type IA and Type V) have been discarded; some enzymes were no longer available in the market and some others were too expensive (moreover, small quantities of powder enzymes are difficult to manage during the preparation process); nevertheless, other enzymes with a low specific activity were supposed to not be very effective. Trypsin from porcine and bovine pancreases could be considered the best compromise considering specific activity, price and ease of use: the final choice fell on trypsin from porcine pancreas. Enzymes were dispersed in an agarose gel loaded with a bile acid soap aqueous solution and buffered at a pH of 8.5. Bile acid soap is a surfactant solution composed of deoxycholic acid deprotonated with triethanolamine. It is especially effective in removing terpen resin such as colophony due to their structural affinity [9,10,11,34,38,39]. In order to use this aqueous solution, it has been verified in the literature that neither deoxycholic acid nor triethanolamine were in concentrations too high to denature the enzymes. Furthermore, the weakly basic pH of 8.5 was optimal to catalyze the action of both enzymes.

Agarose has been chosen as the gelling agent. Agar-agar is now permanently entered into the practice of restoration [40,41,42,43,44,45,46] and agarose is one of the components of agar-agar [47,48,49], exhibiting a lower gelling temperature (around 35 °C) and, with respect to agar-agar (around 43 °C), assuring a safer approach to face the enzymes’ sensitivity to high temperature; enzymes, previously dispersed in aqueous solution at the desired concentration, were added to the agarose solution during its cooling phase, just before gelation.

### 2.2. Model Samples

Model samples have been prepared using different combinations of the materials present in the Vermiglio painting’s beverone, namely lapin skin glue, linseed oil and colophony. Model sample sets were represented by each pure material, binary mixes and a ternary mix for a total of seven sets. Each model sample set consisted of at least eight specimens which were used to test all the possible applications, varying agarose concentrations, time and methods of application (Table 2).

Enzymes were tested only on substrates containing the following potentially hydrolysable materials:

- Trypsin on pure rabbit glue (P); binary mixes (PO and PT); ternary mix (POT);
- Lipase on pure linseed oil (O); binary mixes (PO and OT); ternary mix (POT).

Substrates were prepared applying a given equal quantity of materials on each laboratory glass slide, covering an equal area of 14 cm^2^. In binary mixes the ratio was 1:1 *w*/*w*, while in ternary mixes the ratio was 1:1:1.

Rabbit glue and colophony were dissolved in water and ethanol, respectively; the correct proportions of solvent and solute for each combination were calculated in order to obtain the given ratio at the completion of solvent evaporation (Rabbit skin glue was prepared by dissolving dry glue in water with a ratio of 1:2 by weight. Colophony was dissolved in ethanol 1:1 by weight. In the case of the PT series, a quantity of liquid material equal to two and a half times the quantity of desired dry material was applied to the specimen. Glue was 1:2 in water and colophony 1:1 in ethanol. Five parts in liquid form after solvent evaporation and two parts in dry form with a ratio of 1:1 glue to colophony). In the case of binary and ternary mixes, the different pure materials were previously mixed with a whisk to obtain a fluid and homogeneous compound. In order to minimize errors as much as possible, eight or sixteen samples of each series were chosen among twenty-five replicas of the same substrate. All twenty-five replicas were labelled with the acronym of the series and progressive numbers (e.g., PO1-PO2-PO3…). They were chosen based on the homogeneity of their weight and layer surface. The samples were photographed with macro lenses and with a Dino-Lite microscope (10×–220× magnification; 5 MP) under visible light and UV light to verify the homogeneity within the substrate. For each set, more specimens than necessary were prepared and then the most appropriate specimens for each model sample set were chosen for the gel treatments.

### 2.3. Gels Application

Agarose solutions were prepared through the addition of agarose powder (1 or 2 g) to the DCA-TEA solution and were heated to ~95 °C in a microwave oven. The agarose solutions were then cooled to 42 °C and mixed with the enzyme solutions. The final volume of enzyme-containing melted agarose solutions in DCA-TEA was 50 mL. A lipase enzyme solution was prepared by dispersing 0.25 g of lipase in 15 mL of DCA-TEA heated to 42 °C before being added to the melted agarose DCA-TEA. A trypsin enzyme solution was prepared by dissolving 0.5 g of trypsin in 1 mM of HCl (50 mL), and 1 mL was added to the melted agarose DCA-TEA. Final concentrations in the lipase-melted agarose DCA-TEA solutions were 3500 U/mL lipase and 2 or 4% agarose, whereas in the trypsin-melted agarose DCA-TEA solutions there were 300 U/mL of trypsin and 2 or 4% agarose. Enzyme-melted agarose DCA-TEA solutions were immediately poured directly onto the model sample (fluid application), or casted in silicone molds and placed as pre-formed gels on model samples at the achievement of room temperature (rigid application). Each model sample was treated with 3 mL of enzyme agarose DCA-TEA (Table 3).

Working set conditions were kept constant during the gel applications in order to avoid non-controllable variables and minimize errors. The gel fluid applications were applied on the substrates by means of a polyethylene pipette pre-warmed at 40 °C, just above the agarose gelling temperature.

**Figure 1:**
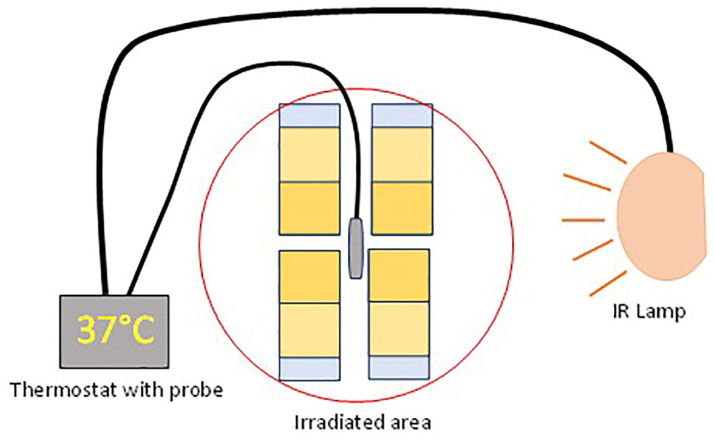
Scheme of the working set.

**Figure 2:**
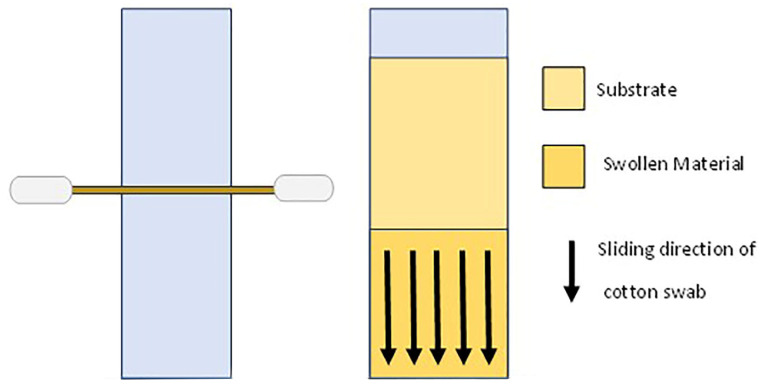
Scheme of swollen material removal after gel application.

Each set was covered by a Melinex sheet to avoid evaporation and drying and immediately incubated for 10 min or 30 min at 37 °C, the optimum temperature for both enzymes, thanks to an IR ceramic lamp connected to a thermostat (Figure 1 and Figure 2).

The gels under testing were applied in two different areas of the glass slide with a double purpose:

1. Calculating the weight loss percentage: after the gel application, swollen material was removed from the samples surface using a two-headed cotton swab, which was bound to a laboratory glass slide of the same thickness as the one used for model samples, and we scrolled it gently five times with the right swab-head and five times with the left one, taking into account the reproducibility of removal of the swollen material (Figure 2). The percentage weight loss value was calculated using the weight value before and after gel application.
2. Collecting samples of swollen materials in order to verify the enzymatic hydrolytic action. For this purpose, after the gel application, swollen materials and gels were collected and submitted to freeze-drying using the Savant concentrator. The FTIR analyses (The study of the peaks was carried out with OriginPro 2021. The procedure followed this process: spectrum smoothing with 9 points and applying the Savitzky-Golay method, baseline correction and subtraction set manually with 15 points, tag of the peaks with minimum height > of 10% maximum height) were focused only on a selection of substrates (specimen treated with 2% agarose-fluid-30 min gels), and we considered them the most promising after the collection of weight loss data.

### 2.4. Evaluation Method and Instrument

An evaluation protocol aimed at assessing the effectiveness was planned, taking into account the published experiences in the field [23,24,25,26,27,28,29]. The protocol consisted of two different steps. In the first phase the percentage of weight loss for every specimen was measured after the gel application. This step was aimed at assessing the ability of the tested formulation in removing the substrate. In the second step FTIR spectra were collected, analyzing materials before and after gel application; then, they were elaborated to underline possible peak changes before and after enzyme application. This second step was aimed at studying the effect of enzymatic hydrolysis over the organic-specific substrate.

#### 2.4.1. Percentage Weight Loss

As a first step, all specimens were weighed with a precision scale (sensitivity 0.0001 g) before and after the treatment. The slides were left in a desiccator for 72 h before measurements were taken in order to remove the moisture released by the gel application.

The percentage weight loss of each specimen was calculated by means of ((M0-M1)*100/M0), in which M0 is the weight before gel application and M1 the weight after gel application.

The data (see Section 3.1) allow for a double comparison:

1. Different gels vs. same substrate material (e.g., eight combinations of trypsin gels on the protein–terpene samples);
2. Same gel vs. different substrate materials (e.g., 2% agarose, fluid, 10 min gel with trypsin or lipase on different substrates).

#### 2.4.2. FTIR Analysis

The substrate material swollen after gel application was collected and analyzed by means of FTIR, comparing the absorption pattern with the untreated material. A Nicolet Nexus spectrophotometer equipped with a Continuμm microscope was used. The transmission spectra were acquired using a diamond anvil cell with MCT detector ranging from 4000 to 650 cm^−1^. Resolution was 4 cm^−1^ and the number of scans was 128. Spectra were elaborated using the software OriginPro 2021. In this second step it was very important to stop the enzyme catalytic reaction on treated substrates at the precise moment the swollen material was collected (freeze-drying process is necessary to avoid that possible enzyme residues in the swollen material continue hydrolysis before the FTIR analysis). For this reason, the treated materials were put into Eppendorf test tubes and instantly freeze-dried using a Savant SC100 SpeedVac Concentrator with an RT100A Refrigerated Condensation Trap (https://www.spectralabsci.com/equipment/savant-sc100-speedvac-concentrator-and-rt100a-refrigerated-condensation-trap/ accessed on 17 December 2023). The freeze-drying process blocked any possible residual enzymatic activity present in the material.

## 3. Result and Discussion

Since the cleaning time and the gel consistency can influence the action of the enzymes and DCA-TEA on the substrates, the cleaning treatment was carried out using different duration times (10 and 30 min), gel application methods (fluid and rigid) and agarose content (2 and 4%). The effect of the cleaning treatments was evaluated by analyzing the model sample weight loss as an index of removal of the layered material and the FT-IR profile changes caused by the treatment to characterize the main material components affected by cleaning.

### 3.1. Specimen Weight Loss

**Figure 3:**
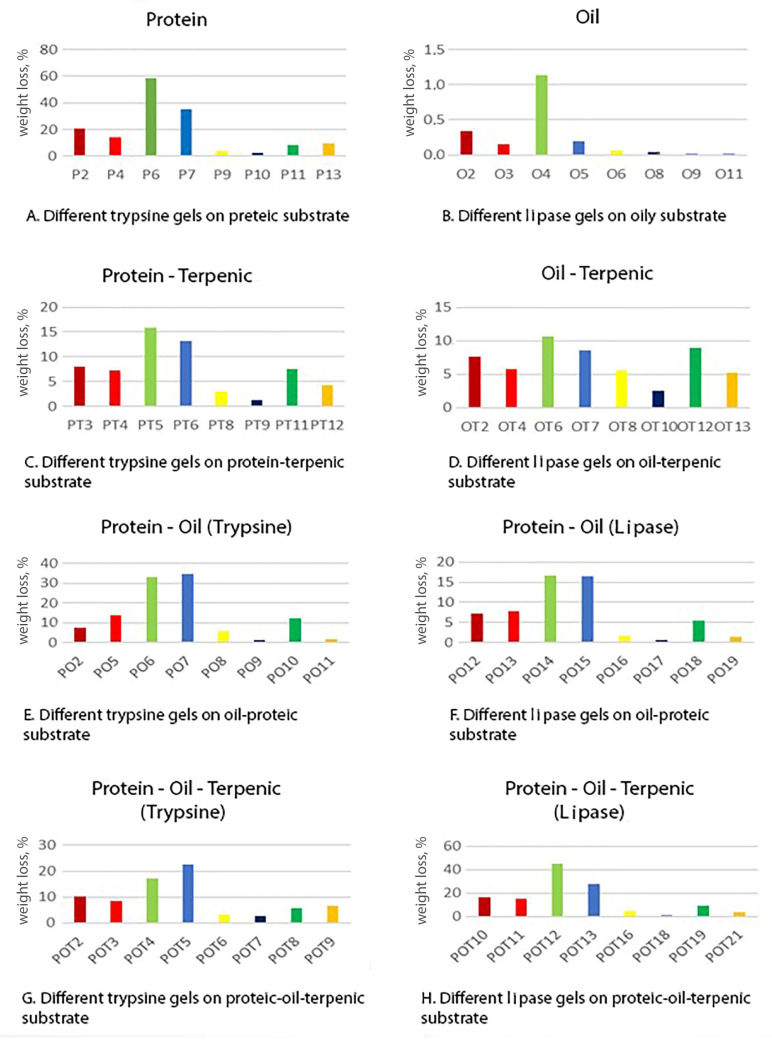
The histograms present the percentage weight loss measured for the different types of gels with the same enzyme on the same substrate. The colors refer to a specific type of gel following Figure 4. Dark red F-2%-10 min; red F-4%-10min; green F-2%-30 min; light blue F-4%-30 min; yellow R-2%-10 min; blue R-4%-10 min; dark green R-2%-30 min; orange R-4%-30 min.

**Figure 4:**
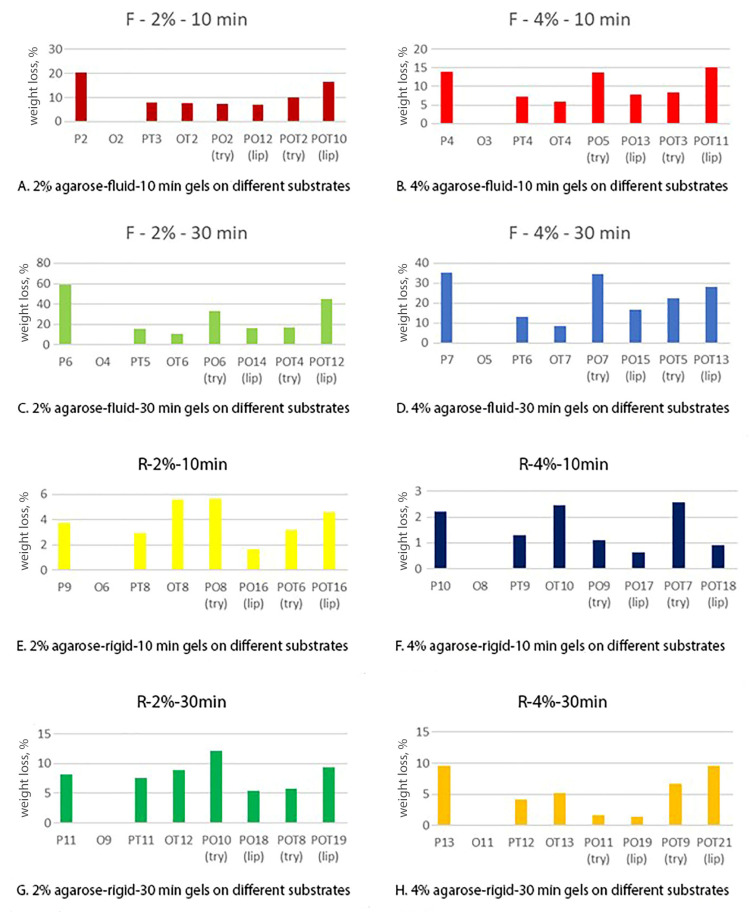
The histograms present the percentage weight loss measured for the same type of gels on different substrates. In the case of the PO and POT substrates, trypsin (try) and lypase (lip) are specified. In each graph, from left to right: Protein–Oil–Protein–terpene–Oil–terpene–Protein–oil (trypsin)–Protein–oil (lypase)–Protein–oil–terpen (trypsin)–Protein–oil–terpen (lypase).

In Figure 3, the weight losses of the model samples due to the interaction of the different tested gels on the same type of substrate are shown; the weight loss is considered proportional to removal effectiveness. In Figure 4, the weight losses due to the use of a given specific gel on different substrates are summarized. The following considerations emerge from a careful examination of the recurring trends:

1. The weight decrease (%) is quite different with regards to the removed materials; however, there is a recurring trend in the two series of histograms. As can be seen from the figure (Figure 3), pure oil displays variations of a few percentage units, therefore, it could be considered not particularly active; most of the mixes are comprised in the range of a 10–40% weight loss; however, the protein substrate shows some peaks up to 60%.
2. For all the substrates, gels applied as a fluid were more effective than the rigid ones. This is probably due to the better gel–substrate contact surface and therefore to a greater release of solution during the application time.
3. There is an obvious linear trend between effectiveness and application time: gels applied for 30 min worked better than the corresponding ones applied for 10 min, as we can expect from the fact that the enzymatic activity normally needs a relatively long time to catalyze the hydrolysis of the material.
4. Gels with the 2% agarose tend to be more effective than 4% ones. These data can be explained by the fact that hydrolytic enzymes work in an aqueous environment and a minor agarose concentration allows the release of a higher quantity of water to the substrate. Furthermore, a minor agarose concentration leads to a large pores size inside the gel network and easier enzyme circulation. The literature confirms that the most suitable percentages for this type of gel range in concentrations of 1.2–2% [36].
5. The removal effectiveness is more influenced by the application time than the agarose concentration. For all substrates, there is a greater difference between two gels with the same agarose concentration and application method, but applied for 10 and 30 min, than between two gels applied in the same way and for the same time, but with different agarose percentages.

**Figure 5:**
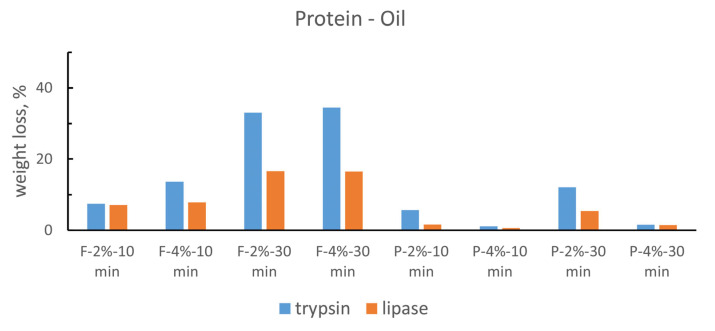
Comparison between trypsin and lipase gel removal action on PO substrate. Values of percentage weight loss.

**Figure 6:**
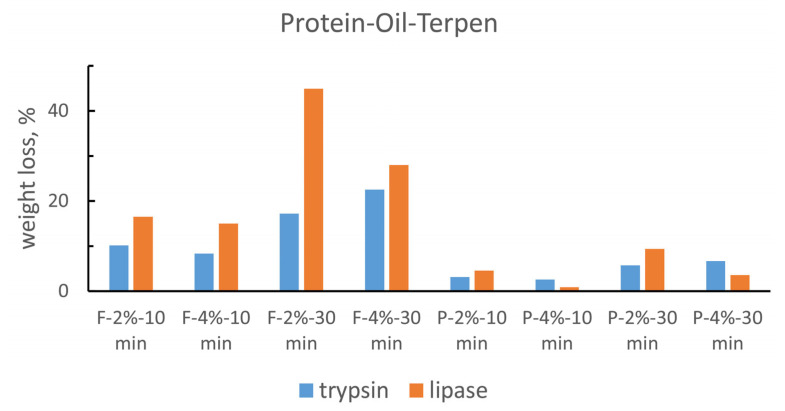
Comparison between trypsin and lipase gel removal action on POT substrate. Values of percentage weight loss.

By also analyzing the second group of histograms (Figure 5 and Figure 6) and comparing the results of the same gel on different substrates, it emerges that trypsin and lipase have a recurring performance when removing all types of materials. In particular, on substrates in which there is the coexistence of protein glue and oil (PO and POT), trypsin is more effective in the first case (PO) than lipase, which has better results in the second one (POT).

This trend can be explained considering that in the PO (glue–oil) substrates the protein component represents 50%, while in the POT mix (glue–oil–colophony), it is only a third; hence, there is a greater amount of trypsin hydrolysable material in the PO substrates than in the POT substrates. Lipase could act better than trypsin on POT due to a greater affinity to resin soap with which it would work in synergy on a substrate that is composed of two thirds lipophilic substances.

This better action could also be due to the method of preparation of the POT substrate, with the colophony previously dissolved in the oil and subsequently mixed with the glue. The oil–resinous blend could be better mixed within the POT substrate and so the lipase, acting on the oil, would also have an action on the colophony by removing a greater amount of material than trypsin gels.

The fact that the lipase acts more effectively on the substrates in which the oil is combined with other materials (OT-PO-POT) than in the application of pure oil could be due to the polymerization and drying mechanisms of the oil. The hydrolysis action of lipase, while taking place mainly at the water/lipid interface [50,51], requires the substrate to be well wet and in close contact with the aqueous solution. The oil is still relatively fresh, but when already polymerized has a compact and continuous structure with a highly hydrophobic behavior that probably does not allow the gel to adhere and wet the substrate sufficiently for enzymatic catalysis.

In the other substrates (PO-OT-POT) the oil has probably failed to polymerize in the same way and has not formed a compact lipophilic barrier; at the same time, the presence of the other more hydrophilic materials, acting with more intense forces of attraction with respect to water, increases the gel/substrate contact surface, thus allowing for a more effective action.

### 3.2. FTIR Spectra Analysis

Following the elaboration process mentioned in Section 2.4.2 FTIR Analysis, spectra of untreated and treated material were processed and percentage values of peak areas were calculated. In this step, only model samples treated with 2% agarose-fluid-30 min gels have been analyzed by FTIR, because they showed the best results according to the “percentage of weight loss” step [52,53,54,55].

FTIR spectra have been considered to assess the peak characteristics of the specific materials used: collagen (from rabbit glue), linseed oil and colophony.

In regards to type I collagen:

(a) The intense band at 1650 cm^−1^ (amide I, C=O peptide bond);
(b) The band around 1540 cm^−1^ (amide II, C-N peptide bond);
(c) A third region between 1400 and 1000 cm^−1^ in which there are some peculiar peaks at 1284, 1240, 1204 cm^−1^ (Amide III) and at 1159, 1062 and 1032 cm^−1^ [45,46,47,48].

In regards to the linseed oil:

(a) The most identifying peaks and bands due to (C-H) CH_2_ stretching, present in the long chains of fatty acids at 2925 and 2850 cm^−1^;
(b) The carbonyl (C=O) attributable to the ester bond between glycerol and fatty acids at 1745cm^−1^ [56,57,58];
(c) Other characteristic peaks at 1658, 1464, 1418, 1240, 1164, 723 cm^−1^ with a further peak at 975cm^−1^ appearing with aging [59].

The colophony spectrum shows a high number of peaks; its identification is hard based only on FTIR, as the terpen components are often very similar to each other. However, the peaks at 2930 (with shoulder at 2950) and 2869 cm^−1^ can be indicated as markers due to the C-H stretching peaks at 1725 and 1695 cm^−1^ (C=O of ketones and carboxylic acids) and several others at 1460, 1385, 1235 and 1174 cm^−1^ as well [60]. The type of spectral processing chosen for this study included, essentially, deconvolution and peak fitting (Before the deconvolution process, transmission spectra were transformed into absorption spectra using the formula A = 2 − log10 (T%), in which A is the absorbance value and T% is the percentage transmittance value). Then, the areas underlying the Gaussians, obtained from the peak fitting process before and after treatment with enzymatic gels, were compared.

#### 3.2.1. Trypsin Gels

Concerning the proteins, the focus has been put on the absorption pattern related to the protein structure changes after hydrolysis. The cleavage of peptide bonds leads to the creation of carboxylate and amino groups, in their ionized form, at the extremities of the two new polypeptide chains produced by peptide bond breaking. This leads to a decrease in the signal referring to amide I (C=O peptide) and to an increase in the one linked to the carboxylate (COO^−^) and amino (NH_3_^+^) groups. This implies a decrease in the area underlying the peak around 1650 cm^−1^ (amide I) and an increase in the one around 1516 and 1400 cm^−1^ for, respectively, NH_3_^+^ and COO^−^. [61] A good method for evaluating variations is aimed at comparing the second derivatives of the spectra before and after hydrolysis. In a paper referring to albumin from bovine serum, the decay of the alpha-helix structure (decrease in the signal at 1654 cm^−1^) in a disordered structure (increase in the signal at 1593 and 1402 cm^−1^) is considered a symptom of successful hydrolysis [62]. Another article proceeds by operating deconvolution and peak fitting peaks in the range of 1700–1600 cm^−1^ before and after hydrolysis; the variation of the contributions given to the amide I band by the various types of conformations present in the secondary structure of the proteins (alpha-helix, beta-sheet, beta-turn, random coil) is achieved. In this case, the curves obtained with peak fitting with peaks centered at 1692, 1677, 1660 and 1643 cm^−1^ decrease, while those at 1617 and 1619 increase [63].

Finally, a deconvolution and peak fitting process (nine-point smooth SG method and straight-line mode baseline subtraction) over a range (1725 to 1375 cm^−1^) was carried out for those substrates containing proteins treated with trypsin gels (P, PT, PO and POT). The hydrolytic action of trypsin should cleave the peptide bonds, varying the secondary structure represented by the band known as amide I (1650 cm^−1^), increasing the intensity of the other peaks referring to disordered polypeptide structures, amino groups (NH_3_^+^) and carboxylates (COO^−^), respectively, at 1610, 1592 and 1402 cm^−1^; particular attention was paid to the latter peak at 1405 cm^−1^, considered to be a reference value together with the amide I one.

Regarding substrates with rabbit skin glue (P), the comparison between the areas underlying the peaks amide I, amide II and the one referable to the absorptions of the carboxylate COO^−^ (1405 cm^−1^) showed no relevant percentage differences between NT and T. A further deconvolution analysis of amide I peak, provided three Gaussian peaks centered at 1690, 1660, 1630 cm^−1^, showing an increase of 1630 cm^−1^, probably indicating some structural changes in the collagen due to enzyme action.

The protein–terpene substrates (PT) did not show significant evidence in peak value variation from untreated to treated specimens. An increase in the carboxylates peak (COO^−^ at 1405 cm^−1^) was observed, which, however, occurred only partially and not unequivocally. There was a very slight decrease in the ratio between the amide I and COO^−^ areas passing from 14.83 to 14.07 (The ratio was made between the average of the three Amide I and COO values—for both NT and T). A more evident change in the ratio of amide I/COO^−^ is visible in the rabbit glue–linseed oil substrates (PO), in which a decrease from 21.08 to 15.09 was observed.

As for the other substrates treated with trypsin gels, for the POT substrate it was difficult to highlight catalytic action. However, observing the peak areas, it can be seen that between NT and T there is a general lowering in the amide I value, a symptom that the protein secondary structure is less present, while COO^−^ value slightly increases. In general, for trypsin gels, clear signs of catalytic action are hardly visible from deconvolution of FTIR spectra; this could be due to the difficulties of reading the FTIR pattern of the proteins and their possible changes.

#### 3.2.2. Lipase Gels

The lipase hydrolytic action on the ester bonds between the glycerol and fatty acids should increase the signal of carboxylic acids coming from the free fatty acids. The signals derived from the C=O bonds of the different functional groups (esters and carboxylic acids) belonging to different molecules are close; hence, deconvolution and peak fitting must be performed to evaluate the contribution of each one within the band at around 1730 cm^−1^. Due to the esters, the C=O occupy the area of the signal around 1767–1740 cm^−1^, while the fatty acids give a signal at lower frequencies, around 1713 cm^−1^ [64]. The literature also proposes to evaluate the appearance of peaks at 1281 cm^−1^ for the C=O of the acid and at 1413 and 941 cm^−1^ for the O-H of glycerol [65].

The ratio of the areas underlying the peaks referring to lipids and proteins as a tool to monitor any changes is suggested as well: for lipids, the peak ranging from 3000 to 2800 cm^−1^ or the carbonyls ester peak (at 1740 cm^−1^), and for proteins the amide I from 1700 to 1600 cm^−1^ can be used [56,57,58,59,60,61,62,63,64,65,66,67,68].

Enzymatic action is expected to act on the triglyceride ester bonds, forming glycerol and fatty acids. This cleavage should vary the contribution of carbonyl around 1750–1690 cm^−1^ [64]. The task is aimed at evaluating the modified contribution of the carbonyl group, taking into account its association with the carboxylic group (in fatty acids) or in esters (tri, bi and mono-glycerides), both before and after the use of enzymatic gels.

Deconvolution and peak fitting were performed in the specified range for each spectrum of those substrates treated with lipase gels (OT, PO, POT) (The spectra were smoothed (smoothed with the nine-point Savitzky–Golay method), taken in the range between 1760 and 1690 cm^−1^ and normalized by subtracting the baseline (straight line with extremes corresponding to the Y values of 1760 and 1690 cm^−1^). We proceeded with the deconvolution and peak fitting using 1740 cm^−1^ for the C=O of the esters, 1710 cm^−1^ for the fatty acids and 1690 cm^−1^ for the carboxylic acids from colophony as the center of the new Gaussians curves). The size of the areas underlying the peaks were correlated to each other (single peak area value divided by the sum of all the peaks areas, multiplied by 100), obtaining the percentage values and comparing the variations.

**Figure 7:**
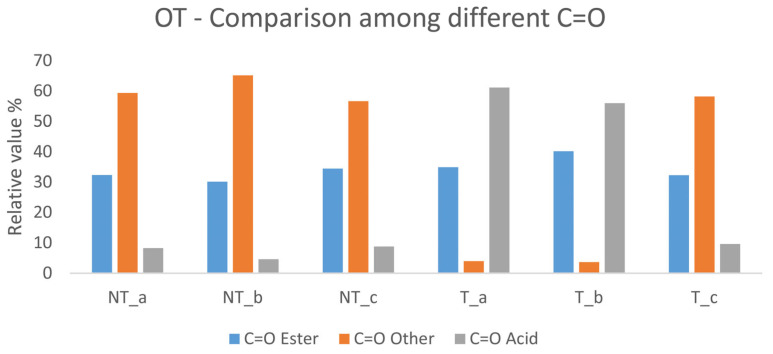
Comparison between the different C=O peak values of the OT substrate before and after lipase gel application.

Lipase gel action seems to be more easily detectable than trypsin action according to the deconvolution analyses and peak fitting. The hydrolysis mechanism involves ester bond, hence the range of 1750 to 1690 cm^−1^, which involves the carbonyl group (C=O) in its various chemical environments (1740 cm^−1^ ester bond; 1710 cm^−1^ fatty acid carboxylic group; 1690 cm^−1^ terpenic carboxylic group), has been analyzed. For the linseed oil–colophony substrate (OT), the percentage values of the areas referring to the fatty acid carbonyl increased sharply in those specimens treated with the enzymatic gel, while those assigned to the C=O of the ester decreased (Figure 7). This can be explained by the hydrolytic action of the enzyme and by the cleavage of the ester bond between the glycerol and fatty acids forming new carboxylic acids.

PO substrates also show the appearance of peaks at 1710 cm^−1^, assigned to the acidic carboxylic group from fatty acids. The ratio between the area of the C=O referring to esters and the one referring to acids decrease from 14.55 to 6.96 (treated–untreated specimen).

**Figure 8:**
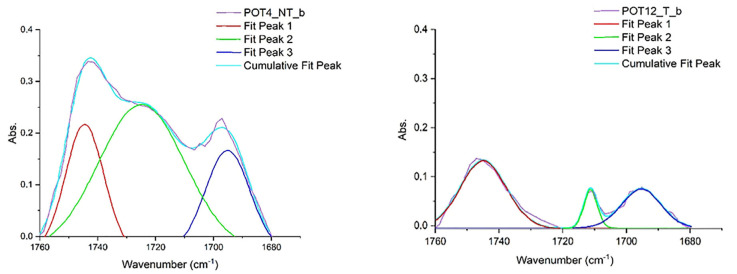
FTIR Spectra of POT substrates. On the left is the untreated sample, on the right the treated sample. Peak 1 refers to C=O group from ester; peak 3 to C=O from acid of colophony. Peak fit 2 changes position from the UT to T sample and it shows the appearance of the C=O signal from the free fatty acid.

As far as the rabbit glue–linseed oil–colophony (POT) substrates are concerned, the lipase gel proved to be more effective in the removal test compared to trypsin gels, and FTIR processing also easily confirmed that enzymatic hydrolysis took place. The percentage values of the areas assigned to the fatty acids C=O increased significantly from NT to T. In Figure 8, deconvolution analyses are reported; a clear separation is visible between the peaks at 1740 and 1710 cm^−1^, with disappearance of the intermediate peak centered at 1720 cm^−1^ [69,70,71,72,73,74,75,76,77,78,79,80,81,82].

## 4. Conclusions

This study is included as a further step in the vast panorama on the use of enzymatic solutions, as well as rigid gels, in artwork conservation [82,83,84,85,86,87,88,89,90,91,92,93,94,95]. Some considerations can be obtained from the analysis of the collected data. First of all, both of the enzymes tested, a trypsin from porcine pancreas and lipase type VII from *C. rugosa*, have been proved by FTIR analysis to be effective in catalyzing the hydrolysis of rabbit glue (cleavage of peptide bonds) and linseed oil (cleavage of ester bonds), respectively. This, however, occurs only when the enzymatic solution is able to sufficiently wet the substrate, creating favorable conditions for the enzyme activity. Fully hydrophobic, compact and homogeneous surfaces such as pure oil coatings do not appear to be hydrolyzed by the lipase-containing enzymatic gels under the conditions tested in the present study. On the contrary, the presence, within the treated substrate, of hydrophilic materials and inhomogeneous surfaces leads to a greater action of the enzymatic gel. The hydrolytic action occurring in the oily–terpene substrate, but not in the oily one, both highly hydrophobic, can be explained by a different mechanism and timing of the oil-drying process, probably influenced by the presence of colophony.

The hydrolytic action on simple and complex materials composed of rabbit glue, linseed oil and colophony in the conditions tested in this study (2% agarose, semi-fluid, 30 min at 37 °C) was confirmed analytically. The reading and processing of FTIR analyses carried out on the materials before and after the application of the enzymatic gels shows, especially in the case of lipase and less so in trypsin, how the enzymes have actually hydrolyzed the substrates. Hydrolysis led to a chemical modification of the materials, the formation of triglyceride-free fatty acids for lipase and the increase in amino acid-related carboxylate groups on the trypsin cleavage sites, which was revealed by IR spectroscopy.

Percentage weight loss results bring further considerations. Among the various types of gels tested, the one that showed the best results on all substrates and with both enzymes was 2% agarose, semi-fluid for thirty minutes. Fluid gels have shown a greater effectiveness than rigid ones and a 30 min application shows a higher action than a 10 min one, confirming the longer times for enzymatic catalysis. The agarose concentration appears to have a minor influence on the results under the experimental conditions tested. The 2% agarose gels show a higher action than those at 4%, probably thanks to a greater substrate water release and easier enzyme circulation within the polymeric network. It also emerged that, under the same conditions, trypsin-containing gels are more effective than lipase one on oily-protein substrates. Conversely, lipase gels have had a greater effect on protein–oily–terpene substrates. The hypothesis is that on the PO substrate, where the protein fraction is more relevant in percentage than in the protein–oily–terpene one, the action of trypsin has a greater chance of catalyzing and swelling. In POT substrates, on the other hand, the higher percentage is represented by hydrophobic materials intimately mixing with each other and interspersing with the protein fraction. These hydrophilic discontinuities could have facilitated the penetration of the enzymatic solution, increased the contact surface of the oily substrate/lipase and favored the hydrolysis which, involving those material that are present in the greatest percentage, led to a greater removal of the lipase gels compared to those of trypsin.

In addition to the results relating specifically to enzymes and enzymatic gels, this paper also provided a valid and reproducible analytical verification method by means of FTIR spectra processing for future studies. The methods of processing spectra, derived from medical and biochemical studies, have been successfully applied to the present experiment. Other enzymes, other substrates, other variables (pH and/or temperatures) or different values of time and agarose concentration could be tested in further research.

Research in this field is moving into its preliminary steps. Moreover, it requires a strong collaboration between conservators, chemists and biologists. The wide possibilities offered by the numerous variables, such as different enzymes added to different natural and synthetic gels, will increase the options offered to conservation projects. It is necessary to highlight that one of the main issues in the field regards the gap still existing between the performance of mock ups and the real cases, even if research has no other path to implement its fallout in good conservation practices.

## Figures and Tables

**Table 1:** Formulation and application methods of the gels tested.

Enzyme	Method of Application	Time of Application	Agarose Concentration	Labelling
Trypsin - Lipase	Fluid	10 min	2%	F-2%-10 min
			4%	F-4%-10 min
		30 min	2%	F-2%-30 min
			4%	F-4%-30 min
	Rigid	10 min	2%	R-2%-10 min
			4%	R-4%-10 min
		30 min	2%	R-2%-30 min
			4%	R-4%-30 min

**Table 2:** Summary of specimen for each substrate.

Specimen Material	Name of Series	Enzyme Tested	N. of Replicas
Rabbit skin glue	P (protein)	Trypsin	8
Linseed oil	O (oil)	Lipase	8
Colophony	T (terpenic)	-	8
Rabbit Glue + Colophony	PT	Trypsin	8
Linseed oil + Colophony	OT	Lipase	8
Rabbit Glue + Linseed oil	PO	Trypsin–Lipase	16
Glue + Oil + Colophony	POT	Trypsin–Lipase	16

**Table 3:** Enzyme gel materials and enzyme concentration.

Lipase Gel	Trypsin Gel
0.25 g of lipase in 15mL of DCA-TEA solution	0.01 g of trypsine in 1mL of 1mM HCl solution
35 mL of DCA-TEA solution with agarose	49 mL of DCA-TEA solution with agarose
Concentration: 5 mg/mL–3500 U/mL	Concentration: 0.2 mg/mL–400 U/mL

## Data Availability

All data and materials are available on request from the corresponding author. The data are not publicly available due to ongoing research using a part of the data.
